# Significant Reduction of Interfacial Thermal Resistance and Phonon Scattering in Graphene/Polyimide Thermally Conductive Composite Films for Thermal Management

**DOI:** 10.34133/2021/8438614

**Published:** 2021-02-23

**Authors:** Kunpeng Ruan, Yongqiang Guo, Chuyao Lu, Xuetao Shi, Tengbo Ma, Yali Zhang, Jie Kong, Junwei Gu

**Affiliations:** ^1^MOE Key Laboratory of Material Physics and Chemistry under Extraordinary Conditions, Shaanxi Key Laboratory of Macromolecular Science and Technology, School of Chemistry and Chemical Engineering, Northwestern Polytechnical University, Xi'an, Shaanxi 710072, China; ^2^Queen Mary University of London Engineering School, Northwestern Polytechnical University, Xi'an, Shaanxi 710072, China

## Abstract

The developing flexible electronic equipment are greatly affected by the rapid accumulation of heat, which is urgent to be solved by thermally conductive polymer composite films. However, the interfacial thermal resistance (ITR) and the phonon scattering at the interfaces are the main bottlenecks limiting the rapid and efficient improvement of thermal conductivity coefficients (*λ*) of the polymer composite films. Moreover, few researches were focused on characterizing ITR and phonon scattering in thermally conductive polymer composite films. In this paper, graphene oxide (GO) was aminated (NH_2_-GO) and reduced (NH_2_-rGO), then NH_2_-rGO/polyimide (NH_2_-rGO/PI) thermally conductive composite films were fabricated. Raman spectroscopy was utilized to innovatively characterize phonon scattering and ITR at the interfaces in NH_2_-rGO/PI thermally conductive composite films, revealing the interfacial thermal conduction mechanism, proving that the amination optimized the interfaces between NH_2_-rGO and PI, reduced phonon scattering and ITR, and ultimately improved the interfacial thermal conduction. The in-plane *λ* (*λ*_||_) and through-plane *λ* (*λ*_⊥_) of 15 wt% NH_2_-rGO/PI thermally conductive composite films at room temperature were, respectively, 7.13 W/mK and 0.74 W/mK, 8.2 times *λ*_||_ (0.87 W/mK) and 3.5 times *λ*_⊥_ (0.21 W/mK) of pure PI film, also significantly higher than *λ*_||_ (5.50 W/mK) and *λ*_⊥_ (0.62 W/mK) of 15 wt% rGO/PI thermally conductive composite films. Calculation based on the effective medium theory model proved that ITR was reduced via the amination of rGO. Infrared thermal imaging and finite element simulation showed that NH_2_-rGO/PI thermally conductive composite films obtained excellent heat dissipation and efficient thermal management capabilities on the light-emitting diodes bulbs, 5G high-power chips, and other electronic equipment, which are easy to generate heat severely.

## 1. Introduction

Flexible electronic equipment are developing rapidly in the directions of high-power, high-density, and high-integration with the advent of the 5G era [1, 2]. The technical integration of high-power chips, wireless charging, and Bluetooth has caused severe heat generation in flexible electronic equipment, seriously affecting the stability, reliability, and service life [3, 4]. Polyimide (PI) is a kind of polymer material with excellent comprehensive properties and various advantages such as low thermal expansion coefficient, high flexibility, high resistance to high and low temperatures, high radiation resistance, and high chemical stability [5–7]. It is widely used in flexible electronic displays, organic light-emitting diodes (LEDs), thin-film solar panels, and other flexible electronic equipment [8, 9]. However, the intrinsic low thermal conductivity coefficient (*λ*) of PI cannot meet the current requirements for efficient and fast heat dissipation of flexible displays, folding screens, and flexible wearable devices, etc. [10, 11].

In recent years, researchers have used graphene [12], carbon nanotubes (CNT) [13], boron nitride (BN) [14], aluminum nitride (AlN) [15], etc. as thermally conductive fillers to prepare thermally conductive PI-based composite films by solution blending followed by blade coating. Wang et al. [16] prepared carbon nitride nanosheets/PI (CNNS/PI) thermally conductive composite films by “solution blending-blade coating” technology. When the amount of CNNS was 20 wt%, the corresponding in-plane *λ* (*λ*_||_) and through-plane *λ* (*λ*_⊥_) of the CNNS/PI thermally conductive composite films reached 2.04 W/mK and 0.32 W/mK, respectively. Song et al. [17] fabricated BN/AlN/PI thermally conductive composite films via “solution blending-blade coating.” The *λ*_||_ and *λ*_⊥_ of the BN/AlN/PI thermally conductive composite films, respectively, reached 4.09 W/mK and 0.44 W/mK when the weight ratio of BN to AlN was 1 : 1 and the total amount was 30 wt%.

However, the reported *λ* values of thermally conductive PI-based composite films are still far from expectation [18, 19]. One of the most important bottlenecks lies in the inherent interfaces between phases and the high interfacial thermal resistance (ITR) [20], which largely limits the efficiency of improvement for *λ* of thermally conductive composite films [21]. The intrinsic mechanism is that the heat flow will be hindered to a certain extent when passing through the interfaces, and severe heat loss will occur, usually manifested as a sudden drop in temperatures at the interfaces [22]. This is mainly because phonon will scatter severely at the interfaces due to vibration harmonic mismatch, acoustic mismatch, and modulus mismatch during the heat transfer process, and the mean free path of phonon will decrease significantly [23], which is not conducive to the rapid and efficient improvement for *λ* of thermally conductive composite films [24]. Therefore, researchers have been struggling to explore suitable methods to effectively improve the interfaces between the polymer matrix and the thermally conductive fillers, so as to reduce the ITR and the phonon scattering at the interfaces [25–27]. Tseng et al. [28] used glycidyl methacrylate (GMA) to graft-modify graphene oxide (*g*-GO) to prepare *g*-GO/PI thermally conductive composite films. When the amount of *g*-GO was 10 wt%, the corresponding *λ*_||_ of *g*-GO/PI thermally conductive composite films was 0.81 W/mK, significantly higher than that of GO/PI thermally conductive composite films (0.32 W/mK) with the same amount of fillers. The main reason is that GMA improved the interfacial compatibility between *g*-GO and PI matrix and reduced the ITR. In our previous work [29], polydopamine (PDA) was performed to functionalize the surface of boron nitride nanosheets (BNNS@PDA), and BNNS@PDA/aramid nanofiber (BNNS@PDA/ANF) thermally conductive composite paper was prepared via vacuum-assisted filtration followed by hot pressing. When the amount of BNNS@PDA was 50 wt%, the *λ*_||_ and *λ*_⊥_ of BNNS@PDA/ANF thermally conductive composite paper reached 3.94 W/mK and 0.62 W/mK, respectively, which were significantly higher than *λ*_||_ (3.33 W/mK) and *λ*_⊥_ (0.52 W/mK) of BNNS/ANF thermally conductive composite paper with the same amount of fillers, due to the hydrogen bonds that improved the interfacial compatibility between BNNS@PDA and ANF.

Although many researchers have tried to improve the interfaces between the thermally conductive fillers and the polymer matrix, most of the studies only use the improvement of *λ* to indirectly characterize the improvement effect of the interfaces [30, 31]. Targeted and detailed researches on ITR and phonon scattering are still in lack [32]. It is acknowledged that Raman spectroscopy can reflect the vibration and rotation of molecules and can reflect the phonon vibration mode, which can be used to characterize the thermal conduction and the degree of phonon scattering at the interfaces [33, 34]. Yue et al. [35] used Raman spectroscopy to study the thermal conduction property of the interfaces between molybdenum disulfide (MoS_2_) and silica (SiO_2_) as well as that between MoS_2_ and graphene, respectively, finding that the thermal conduction property of the interfaces between MoS_2_ and graphene was significantly better than MoS_2_/SiO_2_, because graphene obtains higher *λ* than SiO_2_, and the heat at the interfaces can be transferred more efficiently. Qiu et al. [36] modified gold (Au) nanoparticles on the surfaces of CNTs by chemical vapor deposition (CVD) and used Raman spectroscopy to study the thermal conduction property of the interfaces between CNTs before and after modification. Results showed that the surface modification by Au nanoparticles improved the interfaces between CNTs and effectively reduced phonon scattering at the interfaces.

Currently, Raman spectroscopy is mostly used to study the interfacial thermal conduction properties between inorganics [37, 38]. As far as we know, few researchers have utilized Raman spectroscopy to study the interfacial thermal conduction properties such as ITR and phonon scattering in thermally conductive polymer composite films. This paper is aimed at optimizing the interfaces between reduced graphene oxide (rGO) and PI, and then Raman spectroscopy is innovatively performed to conduct the targeted and detailed investigation on phonon scattering and ITR in the thermally conductive PI-based composite films, revealing the interfacial thermal conduction mechanism. Graphene oxide (GO) was firstly aminated by urea melt (NH_2_-GO), then reduced (NH_2_-rGO), and then NH_2_-rGO/PI thermally conductive composite films were fabricated via “*in-situ* polymerization-blade coating-thermal imidization” technology. X-ray photoelectron spectroscopy (XPS), Fourier transform infrared (FTIR) spectroscopy, thermogravimetric analysis (TGA), Raman spectroscopy, X-ray diffraction (XRD), and atomic force microscope (AFM) were all performed to analyze and characterize the NH_2_-rGO thermally conductive fillers. Raman spectroscopy was performed to characterize the phonon scattering and ITR at the interfaces in the NH_2_-rGO/PI thermally conductive composite films, revealing the thermal conduction mechanism at the interfaces. On this basis, the influences of the amount of NH_2_-rGO thermally conductive fillers, ambient temperature and amination on the *λ*, and mechanical properties and thermal properties of the NH_2_-rGO/PI thermally conductive composite films were analyzed and studied in detail.

## 2. Results and Discussion

**Figure 1:**
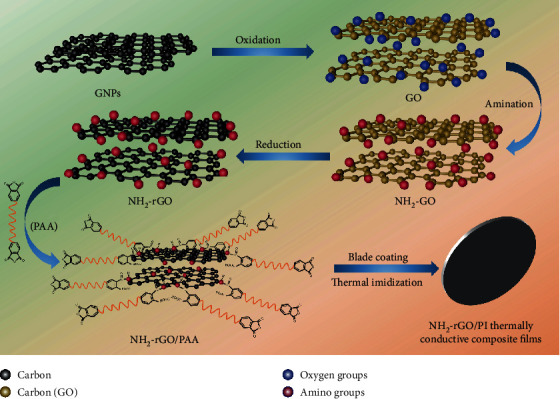
Schematic diagram of preparing NH_2_-rGO thermally conductive fillers and fabricating NH_2_-rGO/PI thermally conductive composite films.

The process of preparing NH_2_-rGO thermally conductive fillers and fabricating NH_2_-rGO/PI thermally conductive composite films is shown in Figure 1. The modified Hummers method was used to oxidize the graphite nanoplatelets (GNPs) to prepare GO, then GO was aminated with urea melt (NH_2_-GO), and then NH_2_-rGO was prepared by thermal reduction. A certain amount of NH_2_-rGO was *in situ* introduced into polyamide acid (PAA), then the amino groups of NH_2_-rGO and the acid anhydride groups at the end of the PAA chains underwent the interfacial chemical reaction, and the two were connected by C-N-C bonds at the interfaces to improve the interfacial properties. Finally, NH_2_-rGO/PAA solution was blade-coated into films and subjected to gradient thermal imidization to prepare the NH_2_-rGO/PI thermally conductive composite films. The experimental details can be found in Supplementary 1.

**Figure 2:**
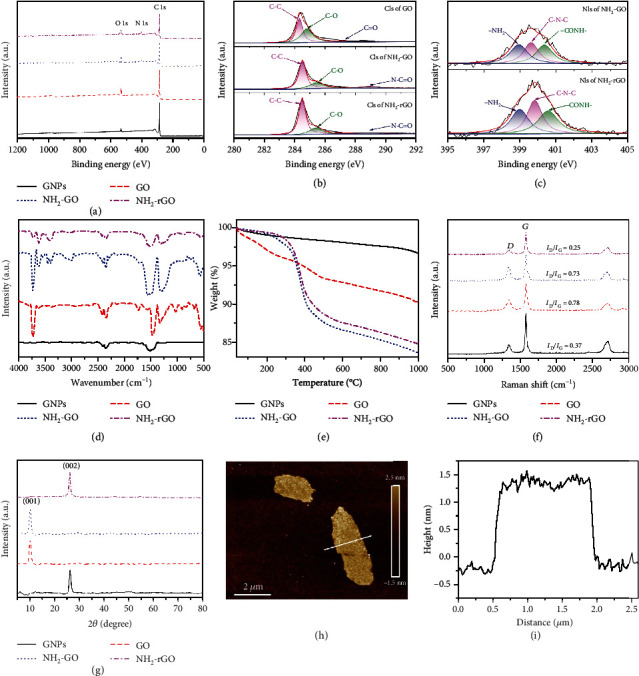
XPS full spectra (a), C 1 s high solution XPS narrow spectra (b), N 1 s high solution XPS narrow spectra (c), FTIR spectra (d), TGA curves (e), and Raman spectra (f), XRD patterns (g) of GNPs, GO, NH_2_-GO, and NH_2_-rGO, AFM image (h) and height-distance curve (i) of NH_2_-rGO.

Figure 2(a) shows the XPS full spectra of GNPs, GO, NH_2_-GO, and NH_2_-rGO. The molar fraction of surface elements and carbon/oxygen (C/O) atomic ratio are shown in Table S1. There are only two kinds of elements, C and O, in GNPs, with the C/O atomic ratio of 26.03. Compared to GNPs, the C/O atomic ratio of GO decreases to 10.49, which is mainly due to the oxidation of GNPs and that more oxygen-containing functional groups (such as hydroxyl, carboxyl, etc.) are introduced. The characteristic peaks of nitride (N) appear in the XPS spectra of NH_2_-GO and NH_2_-rGO, and the C/O atomic ratios are 10.98 and 16.44, respectively. It shows that the amination is successfully performed on GO, and amino groups remain well after thermal reduction. From NH_2_-GO to NH_2_-rGO, the molar fraction of N increases from 3.72% to 5.62%, mainly due to the decrease of oxygen-containing functional groups in NH_2_-rGO [39].

In order to further prove that the N element is chemically bonded to the surface of rGO, the XPS narrow spectra of C 1 s and N 1 s are peak-fitted. Figure 2(b) is the XPS narrow spectrum of C 1 s. The peak of C1s of GO can be fitted to three Lorentz peaks, corresponding to C-C (284.5 eV), C-O (286.5 eV), and C=O (287.3 eV). The C 1 s peaks of NH_2_-GO and NH_2_-rGO can also be fitted to three Lorentz peaks, corresponding to C-C (284.6 eV), C-O (285.4 eV), and N-C=O (289.1 eV). Figure 2(c) exhibits the XPS narrow spectrum of N 1 s. The peaks of N 1 s of NH_2_-GO and NH_2_-rGO can be fitted to three Lorentz peaks, corresponding to -NH_2_ (398.9 eV), C-N-C (399.6 eV), and -CO-NH- (400.4 eV) [40]. The peak fitting of XPS narrow spectrum proves that the amino groups are successfully grafted onto the surface of GO and rGO by chemical bonding.

Figure 2(d) is the FTIR spectra of GNPs, GO, NH_2_-GO, and NH_2_-rGO. GNPs present an absorption peak at 1520 cm^−1^, attributed to carboxyl, because GNPs are slightly oxidized during storage. The newly appeared absorption peaks at 3720 cm^−1^ and 1300 cm^−1^ for GO correspond to hydroxyl and ether bond, respectively, due to the successful oxidation process. After amination, NH_2_-GO presents new absorption peaks at 3610 cm^−1^, 1570 cm^−1^, and 1250 cm^−1^, corresponding to amide, primary amine, and C-N-C groups [39]. It is confirmed that the amino groups have been grafted onto GO successfully. After thermal reduction to NH_2_-rGO, the absorption peaks of ether bond disappear, hydroxyl, and carboxyl are both weakened, and amide, primary amine, and C-N-C groups still maintain, which proves that NH_2_-GO is reduced in oxygen-containing functional groups, and its amino groups are still reserved.

Figure 2(e) exhibits the TGA curves of GNPs, GO, NH_2_-GO, and NH_2_-rGO. There are no obvious thermal weight loss steps in GNPs, and the thermal weight loss is small, which is attributed to the small amount of oxygen-containing functional groups on the surface of GNPs. Compared with GNPs, GO has a greater degree of thermal weight loss, because GO grafts more oxygen-containing functional groups, whose thermal decomposition causes significant weight loss. Both NH_2_-GO and NH_2_-rGO have an obvious thermal weight loss step during 300~500°C, mainly due to the thermal decomposition of amino groups grafted on the surfaces. The thermal weight loss of NH_2_-rGO is smaller than that of NH_2_-GO, due to the removal of some oxygen-containing functional groups of NH_2_-GO during the thermal reduction process. In addition, in the range of 40~140°C, GO shows obvious thermal weight loss while NH_2_-GO does not, which is due to the decomposition of some extremely unstable oxygen-containing functional groups on the surface of GO, and those have been removed during the preparation process of NH_2_-GO [41].

Figure 2(f) shows the Raman spectra of GNPs, GO, NH_2_-GO, and NH_2_-rGO. GNPs, GO, NH_2_-GO, and NH_2_-rGO all have strong Raman D and G peaks at 1341 cm^−1^ and 1580 cm^−1^, which are related to the random vibration of amorphous carbon (sp^3^ hybridized carbon) and in-plane vibration of graphitic carbon (sp^2^ hybridized carbon) [42]. The intensity ratio of D and G peak (*I*_*D*_/*I*_*G*_) is used to characterize the defect density [43]. The higher the *I*_*D*_/*I*_*G*_ value, the more defects in carbon atoms lattice. The corresponding *I*_*D*_/*I*_*G*_ value of GNPs (0.37) is low, indicating that there are only a few defects. The *I*_*D*_/*I*_*G*_ value of GO increases to 0.78, which is attributed to the increase in overall randomness and defects during the oxidation process. After amination, the *I*_*D*_/*I*_*G*_ value of NH_2_-GO (0.73) decreases slightly, basically the same as that of GO, indicating that the amination does not change the structure of GO. After thermal reduction, the *I*_*D*_/*I*_*G*_ value of NH_2_-rGO (0.25) significantly reduces, indicating that thermal reduction restores the graphite structure, reduces the degree of randomness, and effectively repairs some of the defects introduced by the oxidation process.

Figure 2(g) exhibits XRD patterns of GNPs, GO, NH_2_-GO, and NH_2_-rGO. GNPs present a sharp diffraction peak at about 26^o^, corresponding to the (002) crystal plane. In contrast, there is only a sharp diffraction peak at about 10^o^ for GO, corresponding to the (001) crystal plane, due to that the oxidation process disturbs the (002) crystal plane, proving the successful oxidation process. After amination to NH_2_-GO, the XRD pattern shows little difference, proving that the amination does not change the crystal forms of GO. After thermal reduction, the (001) crystal plane disappears and the diffraction peak at 26^o^ corresponding to the (002) crystal plane appears again, proving the successful reduction and that the interplanar spacing is 0.34 nm [44].

Figures 2(h) and 2(i) are the AFM image and corresponding height-distance curve of NH_2_-rGO. The thickness of NH_2_-rGO is uniform, about 1.38 nm on average. As mentioned above, the interplanar spacing is 0.34 nm. Therefore, the number of layers of NH_2_-rGO is about 4. It can be inferred that oxidation, amination, and thermal reduction treatments can effectively exfoliate the GNPs and successfully prepare few-layered NH_2_-rGO thermally conductive fillers.

**Figure 3:**
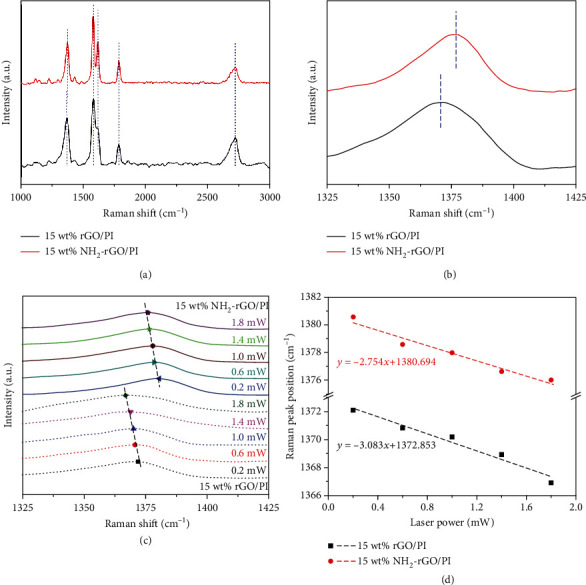
Raman spectra of 15 wt% rGO/PI (a) and 15 wt% NH_2_-rGO/PI (b) thermally conductive composite films with laser power of 1.0 mW. Raman spectra of 15 wt% rGO/PI and 15 wt% NH_2_-rGO/PI thermally conductive composite films with increasing laser power (c). Relationship between Raman peak position of the C-N-C group of 15 wt% rGO/PI and 15 wt% NH_2_-rGO/PI thermally conductive composite films vs. laser power (d).

In order to conduct detailed researches on phonon scattering at the interfaces in NH_2_-rGO/PI thermally conductive composite films, Raman spectroscopy is innovatively utilized.  Figure 3(a) shows the Raman spectrum of 15 wt% rGO/PI and 15 wt% NH_2_-rGO/PI thermally conductive composite films under laser power of 1.0 mW, and Figure 3(b) is a partial view. From Figure 3(a), there are five distinct characteristic peaks of 15 wt% rGO/PI thermally conductive composite films. The characteristic peaks at 1370 cm^−1^, 1580 cm^−1^, 1620 cm^−1^, 1789 cm^−1^, and 2772 cm^−1^ correspond to the axial vibration of the C-N-C groups, the G peak of rGO, the ring vibration of aromatic imide, the asymmetric tensile vibration of the C=O groups, and the 2D peak of rGO [45]. For 15 wt% NH_2_-rGO/PI thermally conductive composite films, the positions of the last four characteristic peaks show no shifts, while the characteristic peak at 1370 cm^−1^ blue-shifts to 1378 cm^−1^ (Figure 3(b)), indicating that the axial vibration of the C-N-C groups in the NH_2_-rGO/PI thermally conductive composite films is enhanced. This is because the NH_2_-rGO fillers and the PI matrix are connected by C-N-C groups, then its vibration is increased. In other words, NH_2_-rGO and PI form chemical bonds (C-N-C groups) at the interfaces, which causes the phonon tangential mode vibration to increase, enhances the “phonon-electron” interaction, therefore causes the blue-shifts of Raman characteristic peak of C-N-C groups, and reduces phonon scattering at the interfaces as well as ITR, and ultimately promotes the improvement of the thermal conduction at the interfaces [46].

Figure 3(c) shows the Raman peak positions of C-N-C groups in 15 wt% rGO/PI and 15 wt% NH_2_-rGO/PI thermally conductive composite films as a function of laser power. The laser power is kept below 2.0 mW to ensure that the samples will not be damaged. Raman characteristic peaks of C-N-C groups in both the 15 wt% rGO/PI and NH_2_-rGO/PI thermally conductive composite films have an obvious red shift with the increase of laser power. When the laser power is increased from 0.2 mW to 1.8 mW, the characteristic peak of C-N-C groups in 15 wt% rGO/PI thermally conductive composite films is red-shifted from 1372 cm^−1^ to 1367 cm^−1^ and that in 15 wt% NH_2_-rGO/PI thermally conductive composite films is from 1380 cm^−1^ to 1376 cm^−1^. This is because as the laser power increases, the temperature at the interfaces of rGO (or NH_2_-rGO) and the PI matrix rises sharply, and the degree of phonon scattering at the interfaces increases sharply, causing the phonon tangential mode vibration to weaken. Next, according to Equation (1) [35], the change trend of Raman peak positions of C-N-C groups in 15 wt% rGO/PI and 15 wt% NH_2_-rGO/PI thermally conductive composite films is linearly fitted with laser power. The fitting results are shown in Figure 3(d).

(1) $$\begin{array}{c} \omega \left(P\right)=\omega_{0}+\chi_{P}P, \end{array}$$

Among them, *ω* is the Raman peak position, *P* is the laser power, *χ*_*P*_ is the first-order power correlation coefficient, that is, the slope of the fitted straight line, and *ω*_0_ is the extrapolated *ω* value when *ω* = 0.

It can be seen from Figure 3(c) that the *χ*_*P*_ in the 15 wt% NH_2_-rGO/PI thermally conductive composite films is -2.754 cm^−1^/mW, and its absolute value is smaller than that of *χ*_*P*_ in the 15 wt% rGO/PI thermally conductive composite films (-3.083 cm^−1^/mW), indicating that the red shift rate of the Raman characteristic peak of the C-N-C groups in the 15 wt% NH_2_-rGO/PI thermally conductive composite films is smaller. This is due to the better thermal conduction property of the interfaces between NH_2_-rGO and PI in the NH_2_-rGO/PI thermally conductive composite films, which can efficiently conduct the highly generated heat by the laser and weaken the phonon tangential mode vibration and the degree of phonon scattering at the interfaces [47].

Figures S1(a)–S1(c) exhibit the scanning electron microscope (SEM) images of cross-sections for pure PI film, 15 wt% rGO/PI and 15 wt% NH_2_-rGO/PI thermally conductive composite films, respectively. The cross-section of the pure PI film shows a typical brittle fracture morphology. With the introduction of thermally conductive fillers (rGO or NH_2_-rGO), the morphology of cross-sections changes, and flakes of rGO or NH_2_-rGO appear around the PI matrix. Moreover, rGO or NH_2_-rGO is basically oriented along the in-plane direction of the PI films. This is due to the blade coating process that promotes the PAA solution to produce the horizontal flow. Driven by the flow forces, rGO or NH_2_-rGO is highly oriented along the in-plane direction of the PI films. Moreover, the agglomeration of rGO is clearly observed while NH_2_-rGO disperses uniformly due to that the amination process improves the compatibility and thereafter enhances dispersion of thermally conductive fillers in the PI matrix. In addition, there are some defects such as holes in the cross-sections of the rGO/PI thermally conductive composite films as the compatibility between rGO and PI is not good. In contrast, for the cross-sections of the NH_2_-rGO/PI thermally conductive composite films, NH_2_-rGO is well compatible with PI and there are no defects or holes, which proves that the amination improves the interfacial compatibility between NH_2_-rGO fillers and PI matrix.

**Figure 4:**
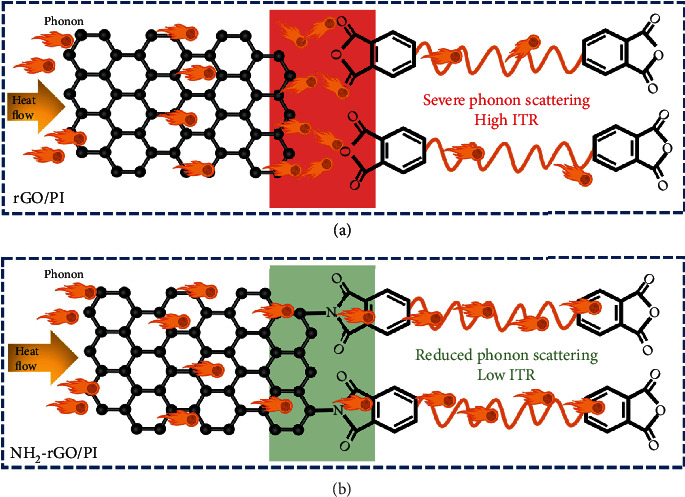
Schematic diagrams of mechanism of interfacial optimization. Schematic diagrams of blocked heat flow, severe phonon scattering, and high ITR at the interfaces in rGO/PI thermally conductive composite films (a). Schematic diagrams of smooth heat flow, reduced phonon scattering, and low ITR at the interfaces in NH_2_-rGO/PI thermally conductive composite films (b).

It is verified that the amination improves the interfaces between NH_2_-rGO and PI and then optimizes the thermal conduction property of the interfaces. The schematic mechanism is shown in Figure 4. For the rGO/PI thermally conductive composite films, there is no connection between rGO and PI. Because of the great difference of the *λ* and surface properties between rGO and PI, severe phonon vibration harmonic mismatch, acoustic mismatch, and modulus mismatch will occur at the interfaces during the heat transfer process, which causes the phonon to be severely scattered interfacially. Therefore, the mean free path of phonon is shortened, and the heat transfer is severely hindered. Finally, the high ITR is formed, which hinders the rapid improvement of the *λ* of the rGO/PI thermally conductive composite films. For the NH_2_-rGO/PI thermally conductive composite films, the amino groups of NH_2_-rGO and the acid anhydride group of PAA undergo the interfacial chemical reaction, and the two are connected by C-N-C bonds with high thermal stability, which exists stably after thermal imidization, so that the NH_2_-rGO and PI are connected through C-N-C bonds, which improves the interfaces between NH_2_-rGO and PI and reduces the phonon vibration harmonic mismatch, acoustic mismatch, and modulus mismatch. The scattering degree of phonon at the interfaces is weakened, the mean free path of phonon is increased, and the ITR is reduced, which is beneficial for the efficient improvement of the *λ* of the NH_2_-rGO/PI thermally conductive composite films.

**Figure 5:**
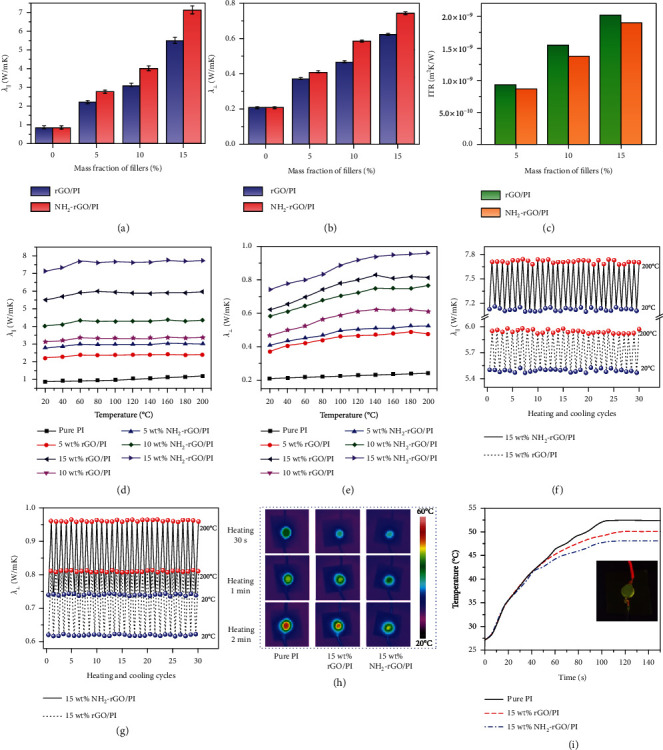
Relationship between *λ*_||_ (a) and *λ*_⊥_ (b) of rGO/PI and NH_2_-rGO/PI thermally conductive composite films vs. fillers' mass fraction. Calculated ITR between fillers and PI matrix in rGO/PI and NH_2_-rGO/PI thermally conductive composite films (c). Relationship between *λ*_||_ (d) and *λ*_⊥_ (e) of rGO/PI and NH_2_-rGO/PI thermally conductive composite films vs. temperature. *λ*_||_ (f) and *λ*_⊥_ (g) of 15 wt% rGO/PI and 15 wt% NH_2_-rGO/PI thermally conductive composite films during 30 heating and cooling cycles. Infrared thermal images of pure PI film, 15 wt% rGO/PI, and NH_2_-rGO/PI thermally conductive composite films (h) and corresponding record of temperature rising ((i), with insert photograph of actual assembly of LED bulb and thermally conductive films).

Figures 5(a) and 5(b) show the relationship between *λ*_||_, *λ*_⊥_ of the rGO/PI and NH_2_-rGO/PI thermally conductive composite films vs. the amount of thermally conductive fillers (rGO or NH_2_-rGO). Both the *λ*_||_ and *λ*_⊥_ of the rGO/PI and NH_2_-rGO/PI thermally conductive composite films increase with the increase of the amount of rGO or NH_2_-rGO. When the amount of NH_2_-rGO is 15 wt%, the *λ*_||_ and *λ*_⊥_ of the NH_2_-rGO/PI thermally conductive composite films are 7.13 W/mK and 0.74 W/mK, respectively, which are 8.2 times *λ*_||_ (0.87 W/mK) and 3.5 times *λ*_⊥_ (0.21 W/mK) of pure PI film, also higher than the *λ*_||_ (5.50 W/mK) and *λ*_⊥_ (0.62 W/mK) of the rGO/PI thermally conductive composite films under the same amount of fillers. It can also be seen that the *λ* of thermally conductive PI-based composite films presents obvious anisotropy as *λ*_||_ is greatly higher than *λ*_⊥_. This is attributed to two aspects. One is that the phonon velocity and mean free path along the molecular chains are higher than those between adjacent chains, and the molecular chains in the PI films tend to orient along the in-plane direction of the PI films, so that the *λ* of pure PI films is anisotropic. The second reason is that the blade-coating process makes the rGO or NH_2_-rGO orient along the in-plane direction of the PI films, so that the number of thermal conduction pathways in the in-plane direction is far more than that in the vertical direction, further making *λ*_||_ higher than *λ*_⊥_. In addition, under the same amount of rGO and NH_2_-rGO, both the *λ*_||_ and *λ*_⊥_ of the NH_2_-rGO/PI thermally conductive composite films are higher than those of the rGO/PI thermally conductive composite films. This is due to the optimization of interfaces between NH_2_-rGO and PI that greatly improves the interface compatibility and reduces the ITR and phonon scattering. In addition, the amination is also conducive to the uniform dispersion of NH_2_-rGO in the PI matrix and is conducive to the formation of more thermal conduction pathways, so that the *λ* of NH_2_-rGO/PI thermally conductive composite films can be quickly and efficiently improved by adding only a few amount of fillers.

In order to further illustrate the effect of the amination on optimizing the interfaces and reducing the ITR, an improved effective medium theory (EMT) model (Equations (2)–(5)) [48] is utilized to calculate ITR between the thermally conductive fillers (rGO or NH_2_-rGO) and the PI matrix in the thermally conductive composite films.

(2) $$\begin{array}{c} \lambda_{||}=\lambda_{m}\frac{2+V_{f}\left(\lambda_{f}/\lambda_{m}\right)\left(1+\langle \cos^{2}\theta \rangle \right)}{2-V_{f}\left(\left(\lambda_{p}H-\lambda_{m}H-R\lambda_{f}\lambda_{m}\right)/\left(\lambda_{f}H\right)\right)\left(1-\langle \cos^{2}\theta \rangle \right)}, \end{array}$$

(3) $$\begin{array}{c} \lambda_{⊥}=\lambda_{m}\frac{1+V_{f}\left(\lambda_{f}/\lambda_{m}\right)\left(1-\langle \cos^{2}\theta \rangle \right)}{1-V_{f}\left(\left(\lambda_{f}H\right)/\left(H+R\lambda_{f}\right)\right)\langle \cos^{2}\theta \rangle }, \end{array}$$

(4) $$\begin{array}{c} \langle \cos^{2}\theta \rangle =\frac{\int \rho \left(\theta \right)\cos^{2}\theta \sin\theta d\theta }{\int \rho \left(\theta \right)\sin\theta d\theta }, \end{array}$$

Among them, *λ*_*m*_ is the *λ* of the polymer matrix, *λ*_*f*_ is the *λ* of the thermally conductive fillers, *V*_*f*_ is the volume fraction of the thermally conductive fillers, *θ* is the angle formed by the orientation direction of the thermally conductive fillers and the in-plane direction of the PI films, *H* is the thickness of the thermally conductive fillers, and *R* is the ITR. When the thermally conductive two-dimensional fillers are completely oriented along the in-plane direction of the PI-based composite films, that is, when *θ* = 0, the model can be simplified as Equation (5):

(5) $$\begin{array}{c} R=\left(\frac{V_{f}\lambda_{⊥}}{\lambda_{⊥}-\lambda_{m}}-\frac{1}{\lambda_{f}}\right)H. \end{array}$$

Figure 5(c) exhibits the calculated relationship between the ITR of rGO/PI and NH_2_-rGO/PI thermally conductive composite films vs. the amount of thermally conductive fillers (rGO or NH_2_-rGO). The ITR of the thermally conductive PI-based composite films increases with the increase of the amount of rGO or NH_2_-rGO, which is due to the increase of the interfacial areas between rGO (or NH_2_-rGO) and PI matrix. In addition, under the same amount of thermally conductive fillers, the ITR in the NH_2_-rGO/PI thermally conductive composite films are lower than those of the rGO/PI thermally conductive composite films. For instance, when the amount of NH_2_-rGO is 15 wt%, the corresponding ITR of NH_2_-rGO/PI thermally conductive composite films is only 1.90 × 10^−9^ m^2^K/W, lower than that in rGO/PI thermally conductive composite films (2.02 × 10^−9^ m^2^K/W).

Furthermore, the influence of ambient temperature on the *λ* of thermally conductive PI-based composite films is also studied. Figures 5(d) and 5(e) show the relationship between *λ*_||_, *λ*_⊥_ of the rGO/PI and NH_2_-rGO/PI thermally conductive composite films vs. ambient temperature. Both the *λ*_||_ and *λ*_⊥_ of the pure PI films increase slowly with the increase of ambient temperature, but the increase rate is small. The *λ*_||_ and *λ*_⊥_ of the pure PI films at 200°C are 1.19 W/mK and 0.24 W/mK, respectively. With the increase of the amount of thermally conductive fillers (rGO or NH_2_-rGO), the increase rate of *λ*_||_ and *λ*_⊥_ with the increase of ambient temperature gradually increases. The *λ*_||_ and *λ*_⊥_ of the rGO/PI thermally conductive composite films at 200°C are 5.95 W/mK and 0.81 W/mK, respectively, and those of the NH_2_-rGO/PI thermally conductive composite films are 7.71 W/mK and 0.96 W/mK, respectively. This is mainly because the increase in the ambient temperature enhances the vibration of the crystal lattice, increases the velocity of the phonon, and then improves their *λ*. Figures 5(f) and 5(g) show the *λ*_||_ and *λ*_⊥_ of 15 wt% rGO/PI and 15 wt% NH_2_-rGO/PI thermally conductive composite films during 30 cooling and heating cycles. At the same temperature during the cooling and heating cycles, the difference in *λ* of the same PI-based composite films in the same direction does not exceed 0.03 W/mK, indicating the excellent stability of *λ* of thermally conductive PI-based composite films during cooling and heating cycles.

Next, the actual heat dissipation test of the thermally conductive PI-based composite films is conducted. Silica gel is used as the adhesive, thermally conductive PI-based composite films are pasted on the bottom of a LED bulb, and the LED bulb will continue to work for a period of time under the voltage of 3 V. An infrared thermal thermography is used to monitor the temperature changes at the surface of the thermally conductive PI-based composite films. Figure 5(h) exhibits the infrared thermal images of pure PI film, 15 wt% rGO/PI, and 15 wt% NH_2_-rGO/PI thermally conductive composite films. Figure 5(i) shows the temperature rise curves of those films, in which the inset figure shows the actual assembly of the device. When the LED bulb starts to work, the temperatures of the thermally conductive PI-based composite films gradually rise. Among them, the heat generates most quickly on the pure PI film and the most slowly for 15 wt% NH_2_-rGO/PI thermally conductive composite films. When the temperatures are stable, the pure PI film has the highest surface temperature (52.4°C), and 15 wt% NH_2_-rGO/PI thermally conductive composite films have the lowest (48.1°C), which further proves that the NH_2_-rGO/PI thermally conductive composite films present more excellent heat dissipation and more efficient thermal management capabilities.

**Figure 6:**
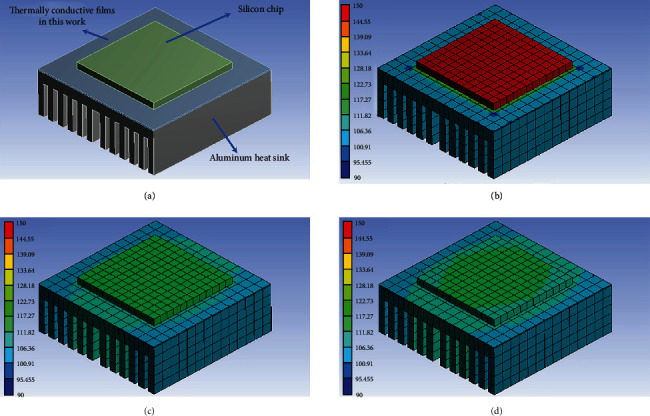
FES model (a). FES results of pure PI film (b), 15 wt% rGO/PI (c), and 15 wt% NH_2_-rGO/PI (d) thermally conductive composite films.

Furthermore, the heat dissipation and thermal management capabilities on 5G high-power chips of pure PI film, 15 wt% rGO/PI, and 15 wt% NH_2_-rGO/PI thermally conductive composite films are simulated through finite element simulation (FES).  Figure 6(a) is the schematic diagram of the FES model, where the silicon chip and the aluminum heat sink sandwich a piece of thermally conductive PI-based composite films as the thermal interface materials, which play the role of heat transfer and protecting the chip. Figures 6(b)–6(d) show the FES results of pure PI film, 15 wt% rGO/PI, and 15 wt% NH_2_-rGO/PI thermally conductive composite films. 15 wt% NH_2_-rGO/PI thermally conductive composite films have relatively better heat dissipation and thermal management capabilities, and the corresponding highest temperature on the surface of the chip (113.0°C) is much lower than those of the pure PI film (148.6°C) and 15 wt% rGO/PI thermally conductive composite films (115.3°C). The above simulation results prove that the NH_2_-rGO/PI thermally conductive composite films present more excellent heat dissipation and efficient thermal management capabilities and can meet the heat dissipation requirements of 5G electronic devices in practical applications.

Figures S2(a) and S2(b) present the tensile stress-strain curves of pure PI film, rGO/PI, and NH_2_-rGO/PI thermally conductive composite films, respectively. Figures S2(c)–S2(f) show the relationship between the tensile strength, elongation at break, Young's modulus, and toughness vs. the amount of thermally conductive fillers (rGO or NH_2_-rGO). The tensile strength of the pure PI film is 70.1 MPa, and the corresponding elongation at break, Young's modulus, and toughness are 6.45%, 1.09 GPa, and 2.56 MJ/m^3^, respectively. With the introduction of rGO or NH_2_-rGO, the Young's modulus of the thermally conductive PI-based composite films gradually increase, due to the effective transfer of part tensile stress in the thermally conductive PI-based composite films to the overall composite networks. But the tensile strength, elongation at break, and toughness of the thermally conductive PI-based composite films gradually decrease, due to that the introduction of rGO or NH_2_-rGO causes some defects in the thermally conductive PI-based composite films. It should be noted that, under the same amount of rGO and NH_2_-rGO, the tensile strength, Young's modulus, and toughness of NH_2_-rGO/PI thermally conductive composite films are all significantly higher than rGO/PI thermally conductive composite films. For example, the tensile strength, Young's modulus, and toughness of 15 wt% NH_2_-rGO/PI thermally conductive composite films reached 35.7 MPa, 2.04 GPa, and 0.322 MJ/m^3^, respectively, which are, respectively, 15.4%, 16.2%, and 11.8% higher than the tensile strength (30.2 MPa), Young's modulus (1.71 GPa), and toughness (0.284 MJ/m^3^) of 15 wt% rGO/PI thermally conductive composite films. This is because NH_2_-rGO and PI matrix are connected by C-N-C bonds, which effectively improves the interfaces. At the same time, NH_2_-rGO, as the dispersed phase, enhances the interaction between the two through the sea-island structure and crack anchor effect, reducing sliding of the PI molecular chains in the NH_2_-rGO/PI thermally conductive composite films during the stretching process, thereby improving the mechanical properties of the NH_2_-rGO/PI thermally conductive composite films [49]. It is clearly shown that the amination can effectively achieve the synergistic improvement of the *λ* and mechanical properties of the NH_2_-rGO/PI thermally conductive composite films, both attributed to the optimization of interfaces.

Figures S3(a) and S3(b) show the differential scanning calorimetry (DSC) and TGA curves of the NH_2_-rGO/PI thermally conductive composite films, respectively, and Table S2 shows the corresponding thermal characteristic data. As the amount of NH_2_-rGO increases, the glass transition temperatures (*T*_*g*_) of the NH_2_-rGO/PI thermally conductive composite films gradually rise. When the amount of NH_2_-rGO is 15 wt%, the corresponding *T*_*g*_ of the NH_2_-rGO/PI thermally conductive composite films increases from 320.2°C of pure PI to 337.7°C. This is because NH_2_-rGO occupies a certain free volume of PI, and the formation of C-N-C bonds between NH_2_-rGO and PI restricts the movement of PI molecular chains, thereby increasing the *T*_*g*_. Meantime, as the amount of NH_2_-rGO increases, the *T*_5_, *T*_30_, and the corresponding heat resistance index (*T*_HRI_) of the NH_2_-rGO/PI thermally conductive composite films gradually increase, that is, the thermal stability gradually increases. When the amount of NH_2_-rGO is 15 wt%, the *T*_5_, *T*_30_, and *T*_HRI_ of the NH_2_-rGO/PI thermally conductive composite films increase from 510.3°C, 588.7°C, and 273.1°C of pure PI to 522.8°C, 605.5°C, and 280.5°C, respectively. The reason is that NH_2_-rGO has good interface compatibility with the PI matrix, and it uniformly disperses in the PI matrix and forms a carbon layer network, thus, effectively prevents the exudation of thermal decomposition products. Moreover, as the temperature rises, the PI matrix absorbs heat and pyrolyzes, and the gas product produced escapes into the boundary layer, blocking the heat entering the interior, forming the thermal blocking effect, which is also beneficial for enhancing the thermal stability of the NH_2_-rGO/PI thermally conductive composite films [50]. It can be deduced that the NH_2_-rGO/PI thermally conductive composite films possess excellent thermal properties and can be used stably for a long period of time under an environment in high temperature.

## 3. Conclusions

XPS, FTIR, TGA, XRD, Raman, and AFM proved that NH_2_-rGO thermally conductive fillers with only a few layers (approximately 4 layers) were successfully prepared. Raman proved that the amination improved the interfaces between NH_2_-rGO fillers and PI matrix and reduced the phonon scattering at the interfaces as well as the ITR. The fabricated NH_2_-rGO/PI thermally conductive composite films present excellent *λ*, ideal mechanical properties, and outstanding thermal stability. When the amount of NH_2_-rGO was 15 wt%, the corresponding *λ*_||_ and *λ*_⊥_ of the NH_2_-rGO/PI thermally conductive composite films at room temperature reached 7.13 W/mK and 0.74 W/mK, respectively, 8.2 times *λ*_||_ (0.87 W/mK) and 3.5 times *λ*_⊥_ (0.21 W/mK) of pure PI film, also significantly higher than *λ*_||_ (5.50 W/mK) and *λ*_⊥_ (0.62 W/mK) of 15 wt% rGO/PI thermally conductive composite films. Calculation based on the EMT model proved that ITR was reduced via amination. Infrared thermal imaging and FES showed that NH_2_-rGO/PI thermally conductive composite films presented excellent heat dissipation and efficient thermal management capabilities on LED bulbs, 5G high-power chips, and other electronic equipment, which are easy to generate heat severely. Meanwhile, the tensile strength, Young's modulus, and toughness of the 15 wt% NH_2_-rGO/PI thermally conductive composite films reached 35.7 MPa, 2.04 GPa, and 0.322 MJ/m^3^, and its *T*_*g*_ and *T*_HRI_ also increased to 337.7°C and 280.5°C from 320.2°C and 273.1°C of pure PI, respectively.

## 4. Materials and Methods

Experimental details including main materials, preparation of NH_2_-rGO thermally conductive fillers, fabrication of NH_2_-rGO/PI thermally conductive composite films, and characterizations can be found in the Supplementary 1.

## Data Availability

The data in this paper cannot be shared at this time as the data also forms part of an ongoing study.

## Supplementary Materials

Supplementary Materials Supplementary 1. S1: Experimental Section: S1.1 Main Materials, S1.2 Preparation of NH_2_-rGO Thermally Conductive Fillers, S1.3 Fabrication of NH_2_-rGO/PI Thermally Conductive Composite Films, S1.4 Characterizations. S2: Supporting Results: Figure S1: SEM images of cross-sections for pure PI film (a), 15 wt% rGO/PI (b), and 15 wt% NH_2_-rGO/PI (c) thermally conductive composite films; Figure S2: Stress-strain curves of rGO/PI (a) and NH_2_-rGO/PI (b) thermally conductive composite films. Relationship between tensile strength (c), elongation at break (d), Young's modulus (e), and toughness (f) of thermally conductive PI-based composite films vs. fillers' mass fraction. Figure S3: DSC (a) and TGA (b) curves of NH_2_-rGO/PI thermally conductive composite films. Table S1: Molar fraction of surface elements and carbon/oxygen (C/O) atomic ratio for GNPs, GO, NH_2_-GO, and NH_2_-rGO.Table S2: Thermal characteristic data of the NH_2_-rGO/PI thermally conductive composite films.
