# Supplementary material for: Significant Reduction of Interfacial Thermal Resistance and Phonon Scattering in Graphene/Polyimide Thermally Conductive Composite Films for Thermal Management
Source: Research (Wash D C). 2021 Feb 23;2021:8438614. doi: 10.34133/2021/8438614 (PMC7931127; doi:10.34133/2021/8438614)
Supplement: Supplementary Materials — Supplementary 1. S1: Experimental Section: S1.1 Main Materials, S1.2 Preparation of NH2-rGO Thermally Conductive Fillers, S1.3 Fabrication of NH2-rGO/PI Thermally Conductive Composite Films, S1.4 Characterizations. S2: Supporting Results: Figure S1: SEM images of cross-sections for pure PI film (a), 15 wt% rGO/PI (b), and 15 wt% NH2-rGO/PI (c) thermally conductive composite films; Figure S2: Stress-strain curves of rGO/PI (a) and NH2-rGO/PI (b) thermally conductive composite films. Relationship between tensile strength (c), elongation at break (d), Young's modulus (e), and toughness (f) of thermally conductive PI-based composite films vs. fillers' mass fraction. Figure S3: DSC (a) and TGA (b) curves of NH2-rGO/PI thermally conductive composite films. Table S1: Molar fraction of surface elements and carbon/oxygen (C/O) atomic ratio for GNPs, GO, NH2-GO, and NH2-rGO.Table S2: Thermal characteristic data of the NH2-rGO/PI thermally conductive composite films. [file 8438614.f1.docx]

**Supplementary Materials**

**Significant Reduction of Interfacial Thermal Resistance and Phonon Scattering in Graphene/Polyimide Thermally Conductive Composite Films for Thermal Management**

Kunpeng Ruan,^1^ Yongqiang Guo,^1^ Chuyao Lu,^2^ Xuetao Shi,^1^ Tengbo Ma,^1^ Yali Zhang,^1^ Jie Kong,^1^ and Junwei Gu^1, *^

^1^MOE Key Laboratory of Material Physics and Chemistry under Extraordinary Conditions, Shaanxi Key Laboratory of Macromolecular Science and Technology, School of Chemistry and Chemical Engineering, Northwestern Polytechnical University, Xi’an, Shaanxi, 710072, P. R. China.

^2^Queen Mary University of London Engineering School, Northwestern Polytechnical University, Xi’an, Shaanxi, 710072, P. R. China.

Corresponding author, E-mail: gjw@nwpu.edu.cn & nwpugjw@163.com (J. Gu)

**S1. Experimental Section**

**S1.1 Main Materials**

Graphite nanoplatelets (GNPs) were purchased from Xiamen Knano Graphene Technology Co., Ltd., China; concentrated sulfuric acid (H_2_SO_4_, 98%) was supplied by Sinopharm Chemical Reagent Co., Ltd., China; phosphorus pentoxide (P_2_O_5_) was provided by Tianjin Kemiou Chemical Reagent Co., Ltd., China; potassium persulfate (K_2_S_2_O_8_) and urea (CH_4_N_2_O) were both purchased from Tianjin Tianli Chemical Reagent Co., Ltd., China; 2, 2’-bis(trifluoromethyl)benzidine (TFDB) and 4, 4’-(hexafluoroisopropylidene)diphthalic anhydride (6FDA) were both provided by Shanghai Aladdin Biochemical Technology Co., Ltd., China; and N, N-dimethylacetamide (DMAc) was supplied by Shanghai Macklin Biochemical Technology Co., Ltd., China.

**S1.2 Preparation of NH_2_-rGO Thermally Conductive Fillers**

In order to effectively prevent the oxidation process from severely destroying and damaging the graphene structure, the traditional Hummers method was improved, and graphene oxide (GO) was prepared by a mild oxidation process. Specifically, 200 mL of H_2_SO_4_, 40 g of K_2_S_2_O_8_, 40 g of P_2_O_5_, and 4.0 g of GNPs were added into a dry three-necked flask, stir evenly and slowly heat it to 80^o^C for 12 hrs. Then a large amount of deionized water was poured for multiple washings till neutrality and GO was prepared. Afterwards, urea melt was used to aminate the surface of GO: a certain amount of GO and urea (1/20, wt/wt) were blended in a dry three-necked flask, heated to 140^o^C to completely melt the urea, and mechanically stirred for 20 hrs. Then a large amount of deionized water was poured to repeatedly wash and finally dried in vacuum to prepare NH_2_-GO. Then an appropriate amount of NH_2_-GO was placed in a tube furnace at argon-hydrogen mixed atmosphere for 2 hrs at 200^o^C, followed by cooling to room temperature naturally to prepare NH_2_-rGO. In addition, in this work, GO was directly reduced to obtain rGO for comparison.

**S1.3 Fabrication of NH_2_-rGO/PI Thermally Conductive Composite Films**

Under nitrogen atmosphere, 0.960 g of TFDB, 1.360 g of 6FDA (TFDB/6FDA, 1/1.02, mol/mol) and 10 mL of DMAc were added into a dry three-necked flask. After mechanical stirring for 10 min in ice-water bath, a certain amount of NH_2_-rGO was added, followed by mechanically stirring in ice-water bath for 5 hrs to prepare the NH_2_-rGO/polyamide acid (NH_2_-rGO/PAA) solution with a certain viscosity. After pouring it on a clean glass substrate, a blade was utilized to coat and to prepare the NH_2_-rGO/PAA composite films. The PI films were dried at 80^o^C for 2 hrs to remove the solvent, and finally the NH_2_-rGO/PI thermally conductive composite films were fabricated by gradient thermal imidization (120^o^C/1 hr+200^o^C/1 hr+250^o^C/1 hr). For comparison, the corresponding rGO/PI thermally conductive composite films were fabricated with the same method.

**S1.4 Characterizations**

Fourier transform infrared (FTIR) spectra of the samples were captured on WQF-510A FTIR equipment (Beijing Rayleigh Analytical Instrument Co., China) to characterize the chemical groups. X-ray photoelectron spectroscopy (XPS) spectra of the samples were investigated on a PHI5400 equipment (PE Co., UK) to characterize the samples to study the element composition and molar fraction of the elements on the surfaces of the thermally conductive fillers. X-ray diffraction (XRD) spectra of the samples were performed on a Shimadzu-7000 type X-ray diffractometer (Shimadzu Co., Japan) to characterize the crystal form of the samples, the incident angle 2θ=5^o^~80^o^, and the step length was 10^o^/min. Thermogravimetric analysis (TGA) of the samples were conducted in nitrogen atmosphere with the heating rate of 10 ^o^C/min by STA 449F3 (NETZSCH Co., Germany) for analyzing thermal stability of the samples. Atomic force microscope (AFM) images of the samples were captured by a Dimension Fast Scan AFM (Bruker Co., USA) to characterize the thickness of thermally conductive fillers. Raman spectra of the samples were collected on an Alpha300R confocal microscope Raman spectrometer (WITec Co., Germany) with a He-Ne laser with a 532 nm laser to study the structural integrity of the thermally conductive fillers, and to characterize the interfacial phonon scattering between the thermally conductive fillers and the polymer matrix. Scanning electron microscope (SEM) images of the samples were collected on a Verios G4 equipment (FEI Co., USA) to analyze the morphology of cross sections for PI films, and the samples were sprayed with gold before testing. In-plane thermal conductivity coefficient (*λ*_∥_) and through-plane thermal conductivity coefficient (*λ_⊥_*) were characterized with Hot Disk TPS2200 thermal constant analyzer by the transient plane heat source method (AB Co., Sweden) according to ISO 22007-2. The samples size was 50 mm×50 mm×100 μm. Infrared thermal images were obtained by a Ti 300 infrared thermography (Fluke, USA).The tensile strength, elongation at break, Young’s modulus and toughness of the films were tested by an Instron Bluehill LE microcomputer-controlled electronic universal testing machine (Instron, USA) in accordance with ISO1184-1983. The samples size was 230 mm×15 mm×100 μm. The corresponding tensile rate was 0.5 mm/min, and each kind of samples should be measured for at least 5 times, and the average value was taken. Differential scanning calorimetry (DSC) curves of the samples were conducted on a DSC1 equipment (Mettler-Toledo, Switzerland) to analyze the *T_g_* with heating rate of 10^o^C/min under nitrogen atmosphere.

**S2.** **Supporting Results**


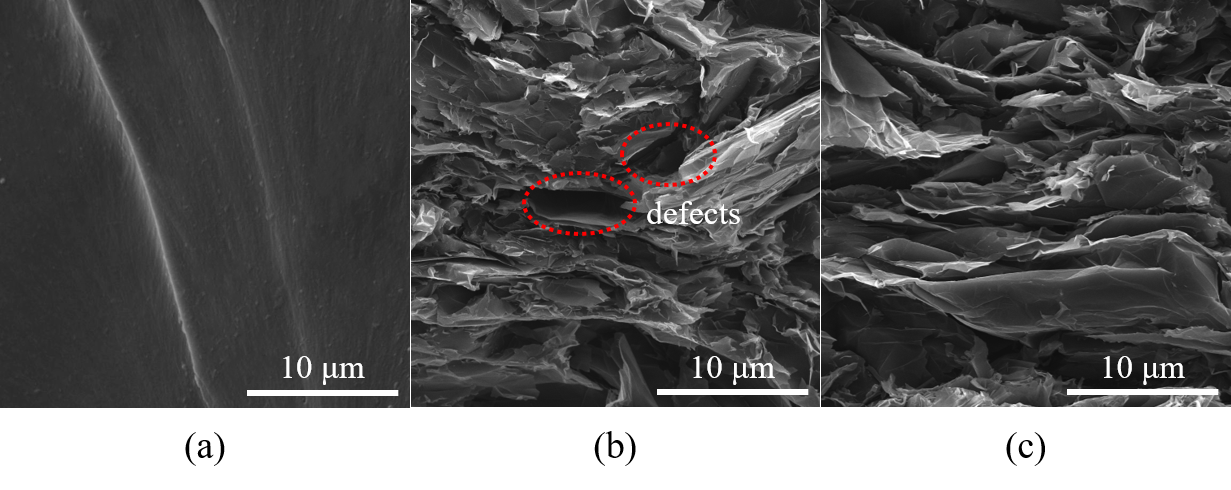


Figure S1: SEM images of cross sections for pure PI film (a), 15 wt% rGO/PI (b) and 15 wt% NH_2_-rGO/PI (c) thermally conductive composite films.


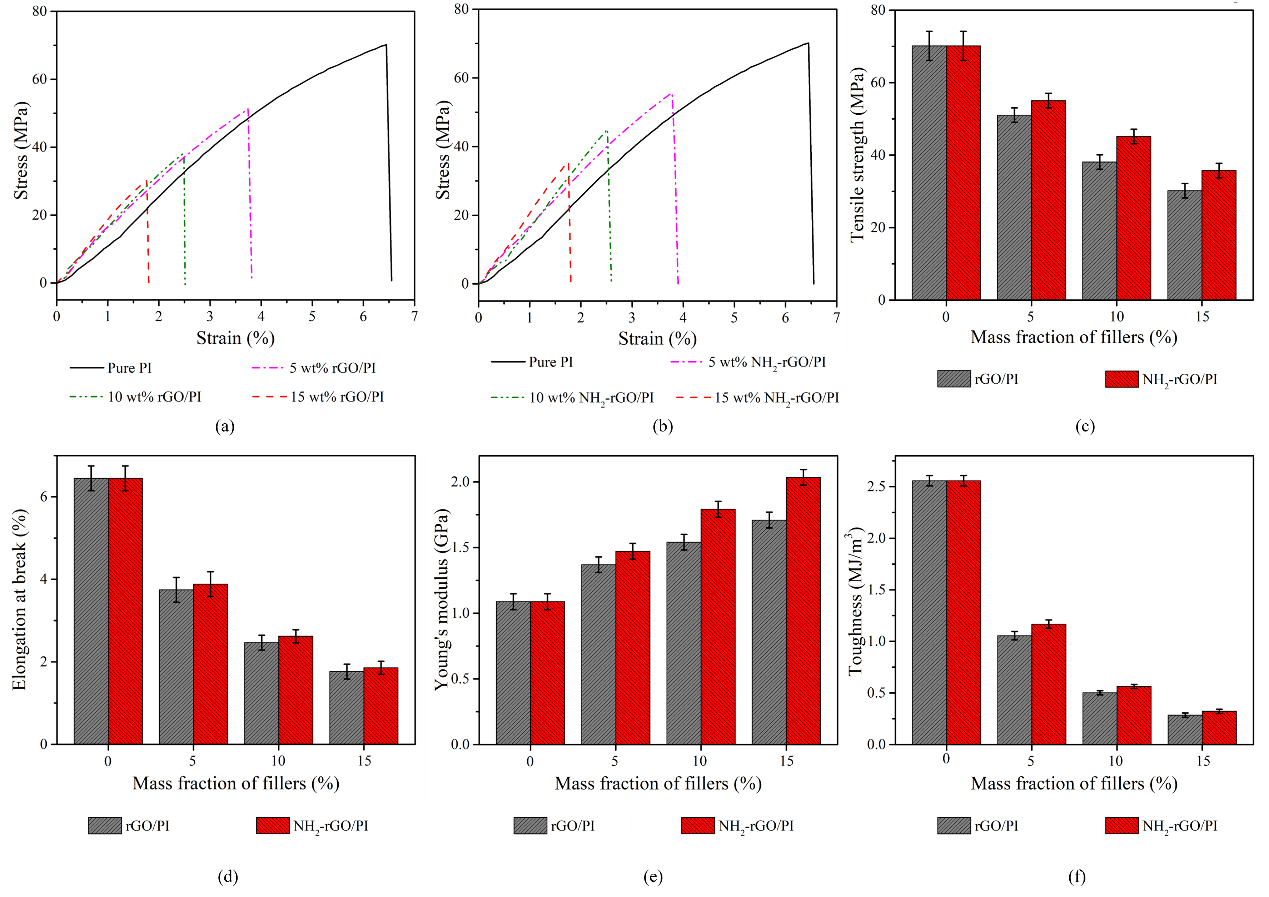


Figure S2: Stress-strain curves of rGO/PI (a) and NH_2_-rGO/PI (b) thermally conductive composite films. Relationship between tensile strength (c), elongation at break (d), Young’s modulus (e), toughness (f) of thermally conductive PI-based composite films *vs* fillers’ mass fraction.


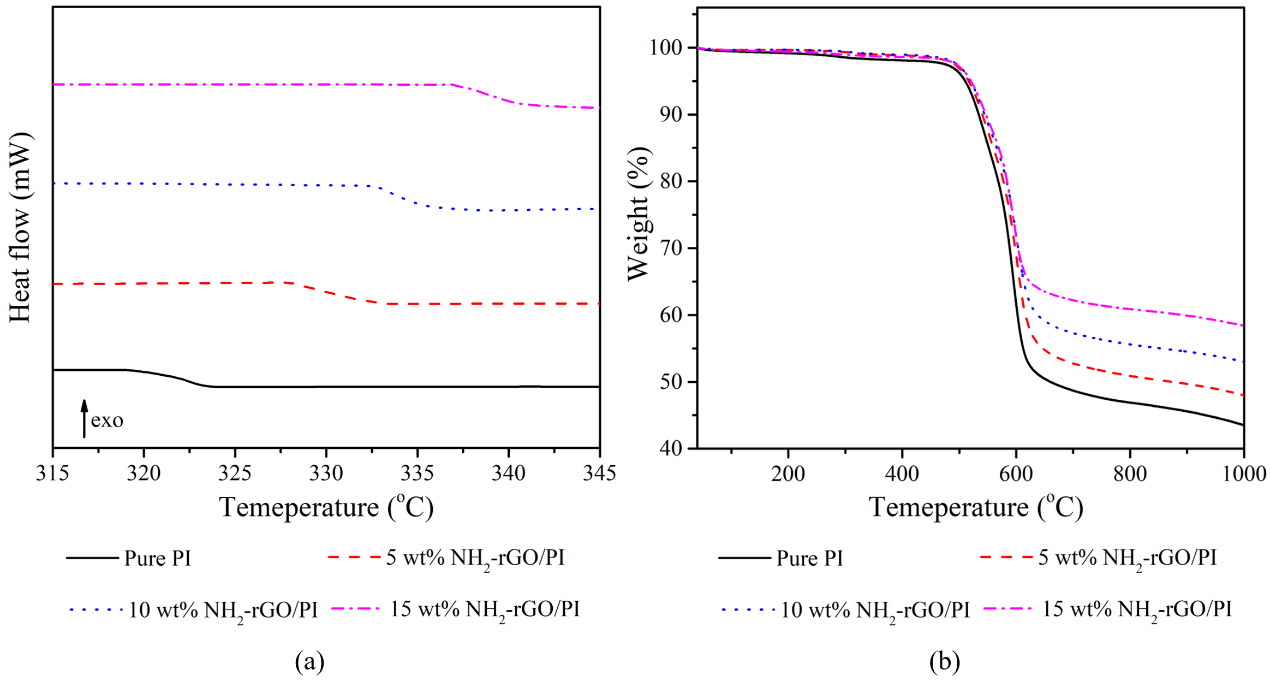


Figure S3: DSC (a) and TGA (b) curves of NH_2_-rGO/PI thermally conductive composite films.

Table S1: Molar fraction of surface elements and carbon/oxygen (C/O) atomic ratio for GNPs, GO, NH_2_-GO and NH_2_-rGO.

|  | Molar fraction (mol%) | | | C/O atomic ratio |
| --- | --- | --- | --- | --- |
|  | C | N | O |  |
| GNPs | 96.30 | - | 3.70 | 26.03 |
| GO | 91.30 | - | 8.70 | 10.49 |
| NH_2_-GO | 88.24 | 3.72 | 8.04 | 10.98 |
| NH_2_-rGO | 88.96 | 5.62 | 5.41 | 16.44 |

Table S2: Thermal characteristic data of the NH_2_-rGO/PI thermally conductive composite films.

| Samples | Weight loss temperature (^o^C) | | | *T_HRI_^*^* (^o^C) | *T_g_* (^o^C) |
| --- | --- | --- | --- | --- | --- |
|  | *T_5_* | *T_30_* | *T_50_* |  |  |
| Pure PI | 510.3 | 588.7 | 660.3 | 273.1 | 320.2 |
| 5 wt% NH_2_-rGO/PI | 516.3 | 598.3 | 873.7 | 277.1 | 328.4 |
| 10 wt% NH_2_-rGO/PI | 520.0 | 603.3 | - | 279.3 | 333.5 |
| 15 wt% NH_2_-rGO/PI | 522.8 | 605.5 | - | 280.5 | 337.7 |

*^*^*$T_{HRI}=0.49\times{[T}_{5}+0.6\times(T_{30}-T_{5})]$, *T_5_* and *T_30_* are corresponding decomposition temperatures of 5 wt% and 30 wt% weight loss, respectively.
